# CcpA Ensures Optimal Metabolic Fitness of *Streptococcus pneumoniae*

**DOI:** 10.1371/journal.pone.0026707

**Published:** 2011-10-21

**Authors:** Sandra M. Carvalho, Tomas G. Kloosterman, Oscar P. Kuipers, Ana Rute Neves

**Affiliations:** 1 Instituto de Tecnologia Química e Biológica, Universidade Nova de Lisboa, Oeiras, Portugal; 2 Department of Molecular Genetics, Groningen Biomolecular Sciences and Biotechnology Institute, University of Groningen, Groningen, The Netherlands; University of Liverpool, United Kingdom

## Abstract

In Gram-positive bacteria, the transcriptional regulator CcpA is at the core of catabolite control mechanisms. In the human pathogen *Streptococcus pneumoniae*, links between CcpA and virulence have been established, but its role as a master regulator in different nutritional environments remains to be elucidated. Thus, we performed whole-transcriptome and metabolic analyses of *S. pneumoniae* D39 and its isogenic *ccpA* mutant during growth on glucose or galactose, rapidly and slowly metabolized carbohydrates presumably encountered by the bacterium in different host niches. CcpA affected the expression of up to 19% of the genome covering multiple cellular processes, including virulence, regulatory networks and central metabolism. Its prevalent function as a repressor was observed on glucose, but unexpectedly also on galactose. Carbohydrate-dependent CcpA regulation was also observed, as for the tagatose 6-phosphate pathway genes, which were activated by galactose and repressed by glucose. Metabolite analyses revealed that two pathways for galactose catabolism are functionally active, despite repression of the Leloir genes by CcpA. Surprisingly, galactose-induced mixed-acid fermentation apparently required CcpA, since genes involved in this type of metabolism were mostly under CcpA-repression. These findings indicate that the role of CcpA extends beyond transcriptional regulation, which seemingly is overlaid by other regulatory mechanisms. In agreement, CcpA influenced the level of many intracellular metabolites potentially involved in metabolic regulation. Our data strengthen the view that a true understanding of cell physiology demands thorough analyses at different cellular levels. Moreover, integration of transcriptional and metabolic data uncovered a link between CcpA and the association of surface molecules (e.g. capsule) to the cell wall. Hence, CcpA may play a key role in mediating the interaction of *S. pneumoniae* with its host. Overall, our results support the hypothesis that *S. pneumoniae* optimizes basic metabolic processes, likely enhancing *in vivo* fitness, in a CcpA-mediated manner.

## Introduction

The survival of bacterial pathogens in different host environments depends largely on the expression of specific virulence factors [1], [2], but also on recognition of nutrients and rapid adaptation to their availability in fluctuating environments. Human bacterial pathogens are heterotrophs that depend on carbon sources to generate energy and catabolic intermediates for growth. In many bacteria, adaptation to changing carbon sources is accomplished through a regulatory mechanism called carbon catabolite control (for reviews see [3], [4]). At the molecular level, carbon catabolite control can be achieved through different regulatory mechanisms, namely transcription activation and repression of genes by global regulators, control of translation by RNA-binding proteins, and allosteric regulation, in response to a preferred carbon source. Overall, these phenomena ensure that carbon sources yielding a maximum profit for growth are utilized first.

In low-GC Gram-positive organisms, such as *Bacillus subtilis* and *Lactococcus lactis,* glucose (Glc) is generally the preferred carbohydrate and the catabolite control protein A (CcpA) is the main transcriptional regulator functioning at the core of carbon catabolite control [5], [6]. CcpA binds to DNA at catabolite-responsive element (*cre*) sites, the location of which, relative to the promoter region, determines whether it acts as a transcriptional inhibitor or activator [5], [6]. The *cre*-binding activity of CcpA is enhanced by interaction with the phosphoprotein HPr, a component of the multi-protein phospho*enol*pyruvate-dependent phosphotransferase system (PTS), when phosphorylated at serine 46 (HPr(Ser-P)) by a fructose 1,6-bisphosphate (FBP) activated kinase [3].

Although the mechanisms by which catabolite control is exerted are among the most studied in model bacteria, a paucity of data hampers our understanding on how human pathogens adapt to different host environments. In the pathogenic Streptococci, increasing evidence supports key roles of CcpA and HPr(Ser-P) [7]–[10]. Notably, CcpA was shown to influence a high percentage of genes in *Streptococcus mutans* (about 9%) and *Streptococcus pyogenes* (up to 20%), including the production of a number of virulence factors [7], [8].

*Streptococcus pneumoniae* is a major cause of life threatening diseases including pneumonia, meningitis and septicaemia, as well as other less severe but highly prevalent infections (e.g. otitis media). Pneumococcal disease is preceded by colonization of the human nasopharynx, *S. pneumoniae*'s natural habitat. It is well established that progression from carriage to pneumococcal invasion is associated with changes in expression of virulence factors, such as capsule and surface proteins [1], [2], but the basic metabolic mechanisms underlying these adaptations remain elusive. Unrelated studies have clearly shown that CcpA influences the expression of genes involved in pneumococcal carbohydrate metabolism [10]–[12]. Furthermore, CcpA has been implicated in the regulation of virulence factors, namely expression of genes in the capsule (*cps*) locus [11] and was proven necessary for colonization of the nasopharynx and survival in the lungs [10]. Curiously, CcpA-independent catabolite repression of metabolic enzymes was a recurrent observation, leading to a view that minimized the role of CcpA as a master regulator of catabolite control in *S. pneumoniae* [10], [11].

In this work, the impact of CcpA on pneumococcal physiology was reappraised by combining genomewide transcriptomics with metabolite analysis of *S. pneumoniae* D39 and its isogenic *ccpA* mutant. Our approach is in line with the current view that true understanding of a microbial metabolism requires quantitative analyses of system components at different cellular levels [13]. A major question was whether *S. pneumoniae* regulates expression of carbohydrate utilization genes and virulence factors in a carbohydrate-dependent, CcpA-mediated manner. Hitherto, the information available has largely been limited to the response of a few specific metabolic genes involved in carbohydrate utilization [10]–[12]. For our global studies, Glc and galactose (Gal) were used as carbon sources for the growth of *S. pneumoniae*. This choice was based on the following: Glc is the most common preferred carbohydrate, and is also found in considerable amounts in the bloodstream and during infection in the respiratory tract of the host [14]. Gal, generally a slowly metabolized non-preferred carbohydrate, was selected among other host-derived carbohydrates as this monosaccharide is one of the prevailing carbohydrates encountered by the pneumococcus in the human nasopharynx (colonization state) [15], [16]. Our data show that CcpA influences the expression profile of central and carbohydrate-specific metabolic genes of *S. pneumoniae* as well as virulence factor-encoding genes in a carbohydrate-dependent and independent manner. These results enhance our understanding of how CcpA-mediated regulation can contribute to the metabolic fitness of *S. pneumoniae* in the host, which further supports the view that virulence and basic microbial physiology are closely intertwined.

## Results

### Growth rates on Glc and Gal are differently affected by CcpA

Hitherto, a systemic appraisal to the role of CcpA in the physiology of *S. pneumoniae* was missing. We constructed a *S. pneumoniae ccpA* deletion-insertion mutant (D39Δ*ccpA*, for details see Material & Methods and Fig. S1A) in the D39 serotype 2 background (Table 1), and determined the growth profiles of the *ccpA* mutant and the wild-type D39 in chemically defined medium (CDM) containing Glc without pH control (Fig. S1B). Under the conditions studied, the maximal biomass reached at time points 8 h and 14 h for strains D39 and D39Δ*ccpA*, respectively, was consistently slightly higher in the absence of CcpA. Loss of CcpA caused a substantial decrease (about 55%, Table 2) of the growth rate in Glc-containing CDM. Importantly, this growth rate defect was fully restored by complementation of a *ccpA* strain (D39Δ*ccpAnisRK*, µ = 0.76±0.00 h^−1^) with expression in *trans* of *ccpA* under the control of the nisin promoter (Fig. S1B). It should be noted that the growth profile of strain D39Δ*ccpAnisRK* closely resembles that of D39Δ*ccpA* (data not shown).

**Table 1:** Bacterial strains, plasmids and primers used in this study.

Strain	Description	Source
***S. pneumoniae***		
D39	Serotype 2 strain, *cps2* (MolGen Laboratory collection)	[58]
D39Δ*ccpA*	D39 Δ*ccpA::spc*; Spc^R^	This work
D39*nisRK*	D39 Δ*bgA::nisRK*; Trim^R^	[50]
D39Δ*ccpAnisRK*	D39 Δ*ccpA::spc* Δ*bgA::nisRK*; Spc^R^, Trim^R^	This work
D39Δ*ccpAnisRK*pNZ[*ccpA*]	D39 Δ*ccpA::spc* Δ*bgA::nisRK* harbouring pNZ[*ccpA*]; Spc^R^, Trim^R^, Cm^R^	This work
***L. lactis***		
MG1363	Plasmid-free *L. lactis* subsp. *cremoris* NCDO712	[59]
**Plasmids**	**Description**	
pNZ[*ccpA*]	pNG8048E carrying *ccpA* downstream of the *nisA* promoter; Cm^R^	This work
pNG8048e	Nisin-inducible P*nisA*, pNZ8048 derivative containing *em* ^R^ to facilitate cloning; Cm^R^, Em^R^	[50]
pNZ8048	Nisin-inducible P*nisA*; Cm^R^	[60]
pORI38	*ori* ^+^ *repA* ^−^, deletion derivative of pWV01; Spc^R^	[61]
**Primers**	**Sequence (from 5′ to 3′)**	**REnz**
ccpA_D39_KO-1	CCAATATTCTTTCAAAACTGG	-
ccpA_D39_KO-2^a^	**TCCTCCTCACTATTTTGATTAG** GGTTACTGTATCATCTGCATTC	-
ccpA_D39_KO-3^a^	**CGTTTTAGCGTTTATTTCGTTTAGT** CATGGTTTGACAGAACGTAGCTC	-
ccpA_D39_KO-4	CCCAGTCGCTCTGGTATCAC	-
ccpA_D39-1^b^	GCCGGTCTCTCATGAATGCAGATGATACAGTAACC	*BsaI*
ccpA_D39-2^b^	GCTCTAGATCGATTCCCTGATTTTTTCTATTTACG	*XbaI*
Spec_Fp	CTAATCAAAATAGTGAGGAGG	-
Spec_Rp	ACTAAACGAAATAAACGC	-
Spd_0420-qRT-1	TGGGAAGGCTTCAAAGG	-
Spd_0420-qRT-2	GGACGAGTGTCCATTGG	-
Spd_0667-qRT-1	TCCATATGCATACGACGC	-
Spd_0667-qRT-2	TGGGATAGATTCTACATCAGC	-
metG-D39-2	TTCTGCCAGCTGGCTTTC	-
metG_D39-1	ATCCGTACAACTGATGAC	-
Spd_0790-qRT-1	GGTGAGGACGGATACTGG	-
Spd_0790-qRT-2	TAACAGTTGCCATACGCTC	-
Spd_1053-qRT-1	GTGCAGATGCTGCAGG	-
Spd_1053-qRT-2	GGCCACGAGTCATATAAGC	-
Spd_1078-qRT-1	TTGACCTTAGTCACGCCC	-
Spd_1078-qRT-2	TTACCTACAAGGTCAAGACGAG	-
Spd_1504-qRT-1	ATGAAACTCAACTTTCGGG	-
Spd_1504-qRT-2	GACATTTTCCAAATCTCGTG	-
Spd_1634-qRT-1	CTGAAACTCTTCGCAAAGAC	-
Spd_1634-qRT-2	TGCGCCGTATGTTCC	-

**Abbreviations:** Spc^R^, spectinomycin resistance; Trim^R^, trimethoprim resistance; Cm^R^, chloramphenicol resistance; Em^R^, erythromycin resistance; REnz, restriction enzyme.

a Overlap with Spc^R^ gene is indicated in bold.

b Restriction enzyme sites are underlined.

**Table 2:** Product yields, carbon and redox balances, growth and energetic parameters, total substrate and pH values determined at the time point of maximal biomass achieved by D39 and D39*ΔccpA* strains cultured on Glc (56±1 mM) or Gal (57±1 mM).

	Glc		Gal	
	D39	D39*ΔccpA*	D39	D39*ΔccpA*
**Product yields**				
Lactate	1.86±0.02	1.71±0.01	1.01±0.04	1.78±0.06
Formate	0.04±0.0	0.20±0.01	0.89±0.00	0.14±0.02
Acetate	BDL	0.12±0.01	0.49±0.01	0.15±0.02
Ethanol	BDL	0.05±0.01	0.45±0.02	0.03±0.00
**q_s_^max^ (mmol g^−1 ^h^−1^)**	31.4±2.8	9.6±2.0	10.0±2.0	9.8±1.5
**µ_max_ (h^−1^)**	0.75±0.03	0.35±0.01	0.27±0.03	0.31±0.01
**Carbon balance** a	95±1	95±1	95±2	96±4
**Redox balance** b	93±1	90±2	96±1	92±2
**Biomass yield (g mol^−1^ of substrate)**	23.1±0.5	30.3±1.1	38.0±1.7	41.8±1.9
**ATP yield (mol mol^−1^ of substrate)**	1.86±0.02	2.00±0.05	2.45±0.00	2.10±0.01
**YATP (g of biomass mol^−1^ of ATP)**	12.4±0.3	15.1±0.2	15.5±0.7	19.8±0.8
**Consumed substrate (%)**	50±1	42±2	26±2	31±0
**pH (growth arrest)**	5.4±0.1	5.5±0.0	5.3±0.0	5.3±0.1

Substrate consumption rate and the specific growth rate are also shown. Values of two or three independent experiments were averaged and errors are reported as ±SD.

a Carbon balance is the percentage of carbon in metabolized Glc or Gal that is recovered in the fermentation products (lactate, formate, ethanol and acetate);

b redox balance is the ratio between [lactate]+2×[ethanol] and 2×[Glc or Gal] multiplied by 100. q_s_^max^ was estimated from a first-order derivative of a polynomial fit of the observed substrate consumption time series. Dry weight (DW) was used as a measure of cell mass. BDL, below detection limit.

To extend our knowledge on the role of CcpA in the physiology of *S. pneumoniae*, growth on galactose was also assessed in batch cultivations as described for Glc (Fig. S1C). On Gal, the maximal biomass produced was also similar in both strains. However, in strong contrast to the effect in Glc-CDM, in Gal-CDM the growth rate of strain D39Δ*ccpA* was slightly higher than that of D39, although this difference was not statistically significant.

Our data show that the effect of CcpA on the growth rate of *S. pneumoniae* is carbohydrate-dependent; CcpA noticeably stimulates fast growth on Glc, but the effect on Gal, if any, is unclear.

### CcpA is a pleiotropic regulator in *S. pneumoniae*

**Figure 1:**
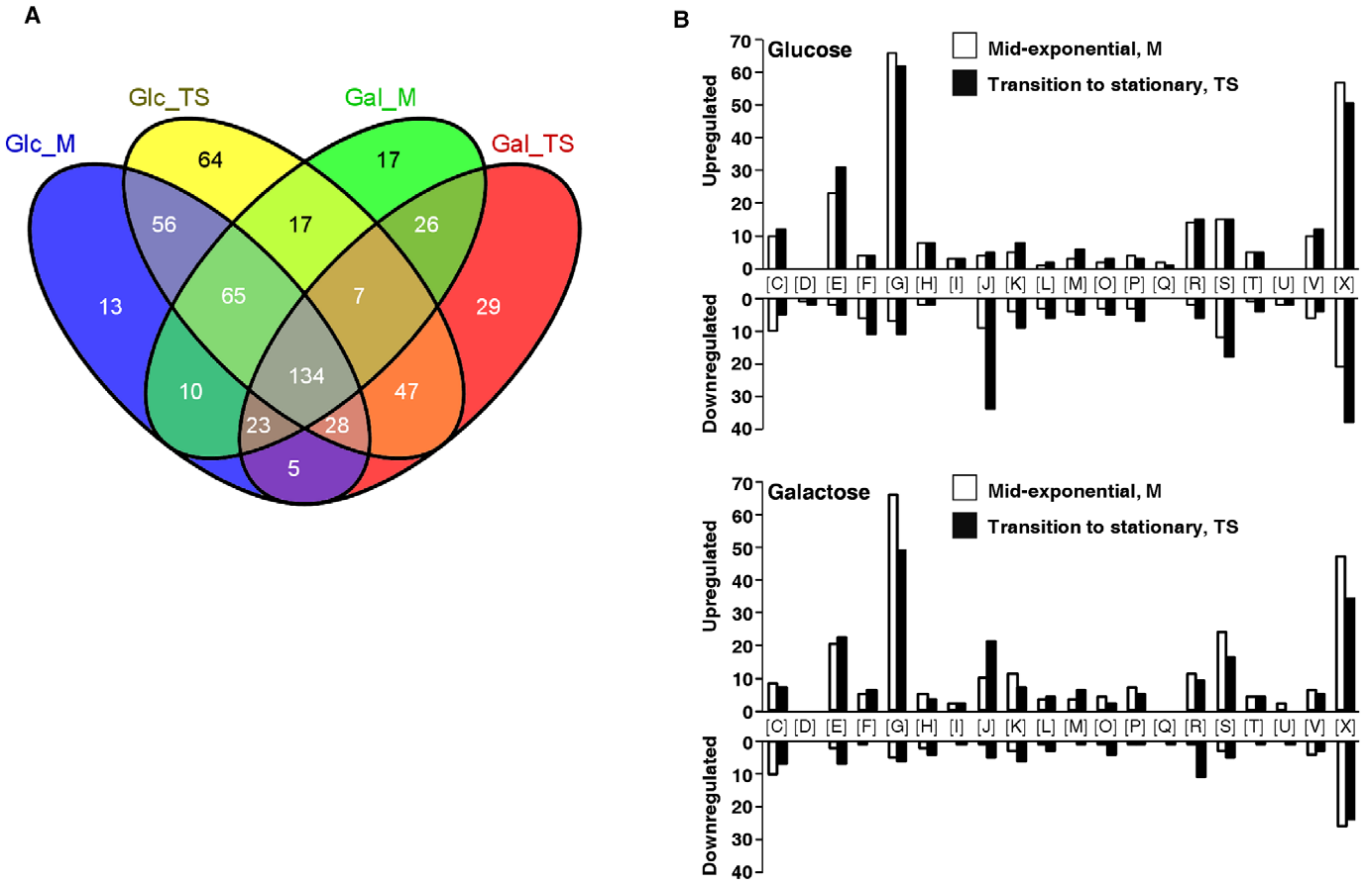
Genes differentially expressed due to inactivation of CcpA. A. Venn diagram of the genes significantly differentially transcribed due to loss of CcpA. Transcript levels in D39Δ*ccpA* were compared to D39 wild-type in Glc_M, Gal_M, Glc_TS, Gal_TS as generated by VENNY (http://bioinfogp.cnb.csic.es/tools/venny/index.html). For all intersections, which are not drawn to scale, the numbers of genes are indicated. The total number of genes influenced in each condition tested was: Glc_M, 334 (15.3%), Glc_TS, 418 (19.2%), Gal_M, 299 (13.7%), and Gal_TS 299 (13.7%) in a universe of 2177 genes in the genome of *S. pneumoniae*. B. Numbers of genes significantly differently transcribed by the *ccpA* deletion in strain D39 ordered by COG categories. White bars represent M phase of growth, black bars TS phase of growth. [C] Energy production and conversion; [D] Cell cycle control, cell division; chromosome partitioning; [E] Amino acid transport and metabolism; [F] Nucleotide transport and metabolism; [G] Carbohydrate transport and metabolism; [H] Coenzyme transport and metabolism; [I] Lipid transport and metabolism; [J] Translation, ribosomal structure and biogenesis; [K] Transcription; [L] Replication, recombination and repair; [M] Cell wall/membrane/envelope biogenesis; [O] Posttranslational modification, protein turnover, chaperones; [P] Inorganic ion transport and metabolism; [Q] Secondary metabolites biosynthesis, transport and catabolism; [R] General function prediction only; [S] Function unknown; [T] Signal transduction mechanisms; [U] Intracellular trafficking, secretion, and vesicular transport; [V] Defense mechanisms; [X] No prediction. The ratio for each gene is >1.5 or <0.66, but >2 or <0.5 in at least one of the four conditions. The number 2177 represents the total number of amplicons analyzed, covering the entire genome of *S. pneumoniae* D39. This number is slightly higher than the number of genes in the genome of D39 (2069), since some amplicons were present in versions for different strains (*i.e.* TIGR4, R6, or D39) or in 2 copies.

To determine the effect of the *ccpA* deletion on the transcriptome of *S. pneumoniae*, the transcriptional profile of D39 wild-type was compared to that of its isogenic *ccpA* deletion mutant at mid-exponential (M) and transition-to-stationary (TS) phases of growth. In the four conditions tested, Glc_M, Gal_M, Glc_TS, Gal_TS, between 14 and 19% of the ORFs in the genome were significantly, differentially expressed in the *ccpA* deletion mutant, of which more genes upregulated than downregulated (Fig. 1, see also Table S1 for an overview). This is similar to *S. pyogenes* (up to 20% genes affected) [8], but higher than *S. mutans*, *L. lactis*, and *B. subtilis* (less than 9% genes affected) [5]–[7]. These findings confirm the role of CcpA as a global regulator with a prevalent repressor function.

The COG category with the highest number of genes affected was carbohydrate transport and metabolism [G], which genes are mainly repressed by CcpA (Fig. 1B). Also, 21 genes encoding (putative) transcriptional regulatory proteins were affected, among which three two-component systems, indicating intertwinement of CcpA with other regulons. In addition, many genes with unknown or unpredicted function, as well as a number of genes within the amino acid transport and metabolism category [E], were among the differentially expressed genes (see also Table S1).

Strikingly, roughly similar numbers of genes were regulated when galactose instead of glucose was used as the carbon source (Fig. 1, Table S1). This means that during growth on the slowly metabolized galactose, carbon catabolite repression by CcpA still takes place. Furthermore, a large part of the genes (up to 44% per condition) differentially expressed were common to all 4 conditions, but carbohydrate and growth phase-specific CcpA-mediated regulation was also in effect (Fig. 1, Table S1).

To firmly confirm our microarray data, changes in transcript abundance of selected genes linked to metabolic and virulence pathways (*lacE*, *galK*, *pyk*, *ldh*, *pfl*, *sodA*, *nanA*), were assessed by qRT-PCR. For all genes tested, expression data obtained by qRT-PCR proved to correlate positively with transcript abundance as measured by microarray analysis (Table S2).

In summary, the role of CcpA as a master regulator in *S. pneumoniae* is undoubtedly substantiated by our genomewide transcriptome analyses. Curiously, not only Glc, but also galactose appears in this study as a repressing carbohydrate in *S. pneumoniae*.

### CcpA influences carbohydrate-specific pathways in carbohydrate-dependent and independent manners

The first step in the metabolism of any carbohydrate is transport across the cell membrane. Genes in 17 out of 29 operons encoding carbohydrate uptake systems are affected in the *ccpA* arrays (Table S3). As for many other categories, several genes encoding carbohydrate transporters were not only strongly repressed by CcpA on Glc, but also on Gal. *ManLMN* (SPD_264-2) codifying the mannose/glucose-PTS, a predominant Glc transporter in *Streptococcaceae* [3], was among them. Carbohydrate-specific CcpA-mediated regulation was also in effect: SPD_0661, a Glc-family PTS system, was exclusively repressed in Gal-CDM. This gene is predicted to be a direct target of CcpA due to the presence of a *cre* site in its promoter region. Another PTS operon (SPD_0559-0562), which also contains a *cre* site in its promoter region, was strongly negatively regulated on Glc, but only mildly on Gal. Curiously, the latter PTS system has been previously postulated to transport Gal [12].

**Figure 2:**
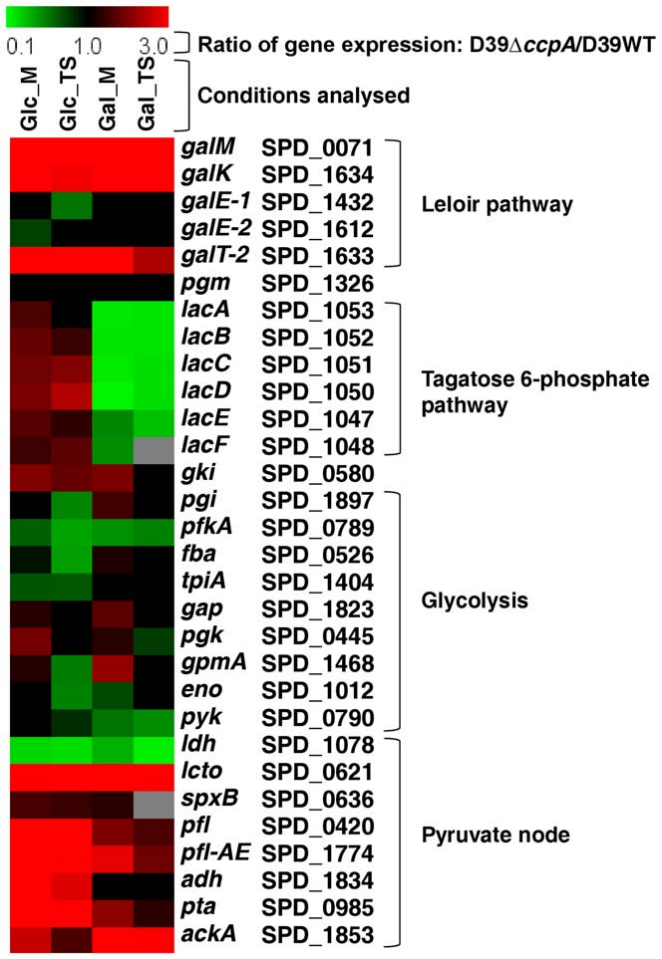
Visual representation of the effect of CcpA on the transcription of genes involved in key basic metabolic processes. Ratio's of genes in glycolysis, the Leloir and tagatose 6-phosphate pathways, and the pyruvate node, in all four conditions analysed in this study (Glc_M, Gal_M, Glc_TS, Gal_TS) are depicted. On top of the figure, a colour scale is given for the ratio of the expression in the *ccpA* mutant over that in the wild-type strain. Thus, red means repression by CcpA (upregulation in the microarray analysis) and green means activation by CcpA (downregulation in the microarray analysis). For each gene the D39 *locus tag* and the gene name is given on the right. No cut-off value was applied for the expression ratios of the genes given in the figure. Genes were considered significantly changed when having a Bayesian *p*-value and FDR meeting the criteria as outlined in the Materials & Methods. Genes that did not meet these criteria were given a ratio of 1.0 (black colour), meaning no significant change in expression. See Fig. 5 for an overview of these pathways in *S. pneumoniae* D39.

A genome survey of *S. pneumoniae* indicates that it possesses both the Leloir and tagatose 6-phosphate Gal catabolic pathways. Interestingly, the Leloir genes were repressed by CcpA independently of the carbohydrate, whereas the tagatose 6-phosphate pathway was weakly repressed by CcpA on Glc and strongly activated on Gal (Fig. 2). Also of note was the observed induction of the tagatose 6-phosphate pathway (10- to 12-fold higher) and the Leloir genes *galK* and *galT-2* (25 times higher) by Gal, regardless of CcpA (Table S4). As for the *lac* genes, other intracellular carbohydrate-specific metabolic steps were subjected to CcpA-mediated regulation in a carbohydrate dependent manner (Table S1). For example, a putative 1-*pfk* (SPD_0772) and an upstream *lacR* were upregulated in the *ccpA* mutant only on Gal.

### *Cre* site prediction in the genome of *S. pneumoniae*

To discern between direct and indirect CcpA dependent effects, an *in silico cre* site prediction was performed in the genome sequence of D39 (for details see Materials & Methods), yielding 90 predicted *cre* sites that match with the genes differentially expressed in the *ccpA* DNA microarray data in at least one condition (see Tables S1, S5 and S6). A consensus *cre* is shown in Fig. S1A. A rough estimation of operons in *S. pneumoniae* using Genome2D [17], based on sizes of intergenic regions and gene orientation, resulted in the division of the differentially expressed genes in 215 transcriptional units. This means that approximately 42% of the promoters regulated in the arrays have a *cre* site and may therefore be directly regulated by CcpA. When considering only the genes with a *cre* site in their promoter region, 68 genes (76%) have a *cre* site in a position that agrees with the observed regulation in the DNA microarray analysis (see Table S1). For the remaining genes, either the promoter could not be predicted or regulation was weak and opposite when comparing the different conditions. Notably, there were many genes with a *cre* site in their promoter region that were not affected by the *ccpA* deletion (Table S6). On the other hand, the expression of some genes was highly changed, although a *cre* site was not found. This suggests indirect regulation of these genes, possibly by regulators that are influenced by CcpA.

### CcpA is by large an activator of glycolysis and influences genes involved in fermentation

The role of CcpA in controlling expression of genes involved in central carbon metabolism is well established [5], [6], [8]. Despite its prevalent repressor function, in *S. pneumoniae* CcpA was shown to activate several glycolytic genes (Fig. 2). The larger positive effects were observed for the key glycolytic gene *pfkA* (6-phosphofructokinase) and in *ldh* (lactate dehydrogenase), but activation of *pyk* (pyruvate kinase) was also considerable, which strongly suggests a predominant role of CcpA in assuring the efficient conversion of carbohydrates to lactate in this bacterium.

In line, CcpA was in all instances a repressor of *pfl* (SPD_0420, pyruvate formate-lyase), which encodes a key activity that directly competes with lactate dehydrogenase for pyruvate, as well as of *pfl-AE* (pyruvate formate-lyase activating enzyme) (Fig. 2, Table S1). Carbohydrate-independent CcpA-dependent regulation of genes linked to the pyruvate node has been previously reported [18]. Moreover, the genes in the pathway leading to acetate, *pta* (SPD_0985, phosphotransacetylase) and *ackA,* (SPD_1853, acetate kinase) were upregulated in the mutant. Curiously, *adh* (SPD_1834, alcohol dehydrogenase) was also repressed by CcpA, but only in Glc containing medium. Moreover, in the parent strain D39 the expression of *pfl*, *pfl-AE* and *adh* was induced by Gal, as denoted by the decimal fold change ratio Glc/Gal (Table S4). Overall, the expression pattern of genes involved in fermentative steps (activation of *ldh* and repression of mixed-acid fermentation genes) (Fig. 2) strengthens the view that the pneumococcal CcpA promotes homolactic fermentation.

### Loss of CcpA changes the fermentation profiles of D39 on Glc and Gal

The dissimilar growth of D39 and D39Δ*ccpA* and the resulting expression profiles prompted us to perform an in-depth characterization of the fermentation patterns on Glc and Gal.

#### Substrate consumption and end-products

**Figure 3:**
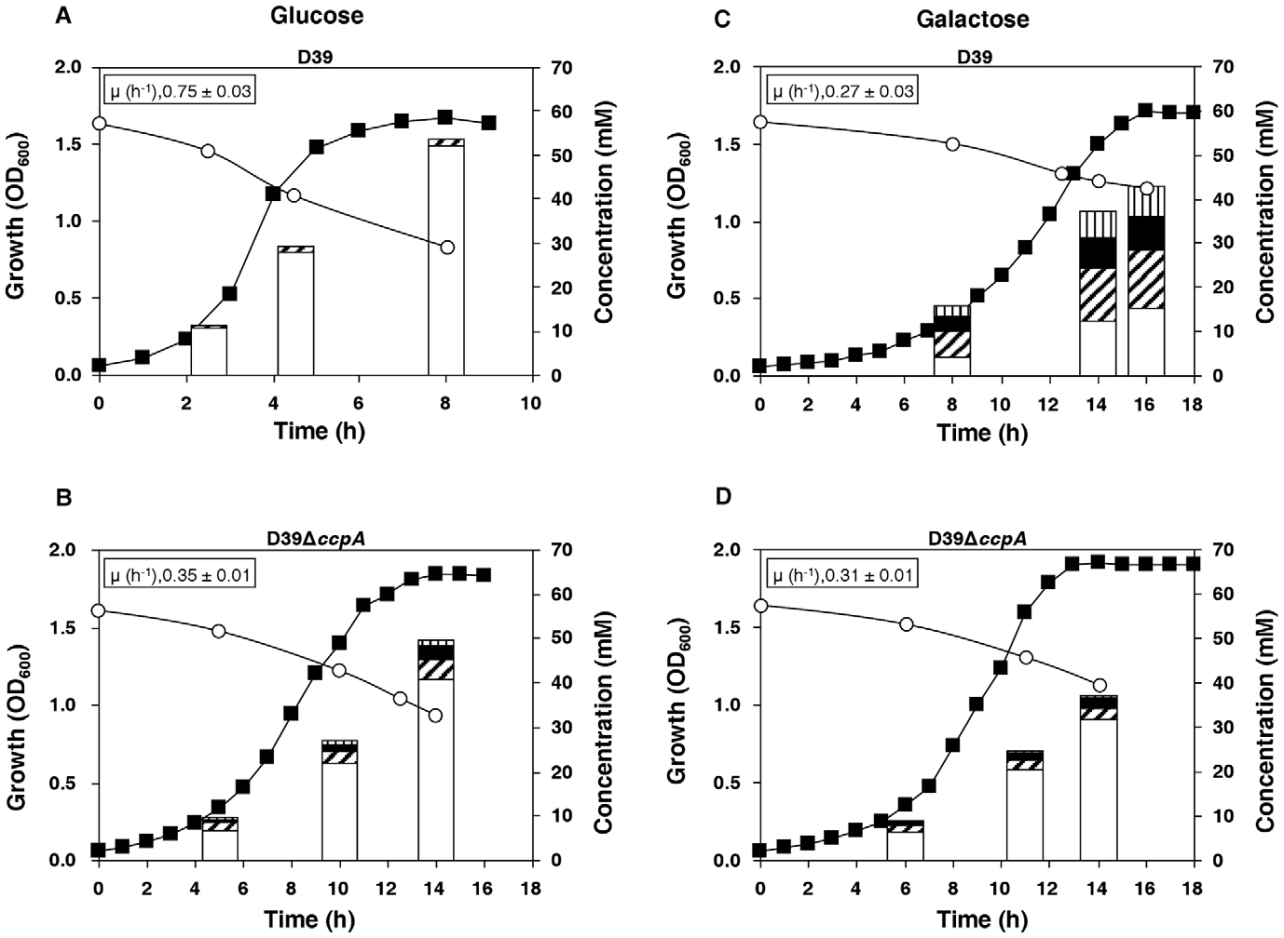
Fermentation profiles of D39 and D39Δ*ccpA* on Glc and Gal. Growth curves, substrate consumption and end-products formed by the D39 (A and C) and D39*ΔccpA* (B and D) strains growing on Glc (A and B) or Gal (C and D). Culture supernatant samples for substrate and end product analysis by HPLC and/or ^1^H-NMR were harvested for each of the conditions in the mid-exponential, transition-to-stationary and growth arrest (maximal biomass) time points of the respective growth curves (bars in the plots). To calculate end-product concentrations, values of at least two independent experiments were averaged and the error was below 7% for major products (>2 mM) and 25% for minor products (<2 mM). Initial concentrations of Glc and Gal were 56±1 mM and 57±1 mM, respectively. Time points in substrate consumption curves are averages of at least two independent experiments and the error is below 5%. Symbols: (○), substrate consumption; (▪), growth curve; white bars, lactate; hatched bars, formate; black bars, acetate; stripped bars, ethanol. Growth curves as in Fig. S1, except that OD_600_ scale (y-axis) is decimal.

The pneumococcal strains were grown as above. Substrate consumption and concentrations of end-products are shown in Fig. 3. During growth on Glc strain D39 showed a typical homolactic behaviour, the major end-product being lactate, which reached a concentration of 52±1 mM at the time of growth arrest (maximal biomass), accounting for 93% of the Glc consumed (Table 2); formate was also formed in minor amounts (Fig. 3A and Table 2). Loss of CcpA caused a shift towards a more mixed acid fermentation: a 5-fold increase in the yields of formate, as well as formation of acetate and ethanol, were accompanied by a decrease on lactate production, which accounted for 85% of the Glc consumed as compared to 93% in D39 (Fig. 3B and Table 2). Hydrogen peroxide (H_2_O_2_), a known minor end-product of streptococcal aerobic metabolism, was also detected. In the wild-type, the maximal concentration reached was about 0.04±0.002 mM, while in the mutant this concentration was *circa* 7-fold enhanced. The low concentrations measured most likely reflect a limitation in oxygen availability due to the nearly anaerobic conditions used for growth. At the time point of growth arrest, the *ccpA* mutant had consumed 8% less Glc, and the maximal consumption rate was about 30% of the wild-type level (Table 2). The observed decrease in Glc consumption was paralleled by the reduction in growth rate, which was 53% lower in D39Δ*ccpA.*

In Gal-containing medium strain D39 displayed a mixed-acid fermentation pattern with lactate, formate, acetate and ethanol as end-products (Fig. 3C). At the time of growth arrest, lactate and formate accounted each for about 50% of the Gal consumed (Table 2). Formate, acetate and ethanol were produced in a 2∶1∶1 ratio, denoting pyruvate formate-lyase activity. This pronounced shift to mixed acid fermentation in strain D39 might be partially explained by alleviation of the CcpA-mediated repression of *adh, pfl* and *pfl-AE* during growth on Gal (Table S4). The molecular mechanism underlying the partial de-repression of the mixed-acid fermentation genes by CcpA on Gal is not understood.

Unexpectedly, in this carbohydrate inactivation of CcpA resulted in a more homolactic profile: the yields of formate, acetate and ethanol decreased, whereas the yield of lactate increased (Fig. 3D, Table 2). As opposed to Glc-CDM, on Gal the H_2_O_2_ produced was slightly higher in the wild-type (0.31±0.02 mM) than in the *ccpA* mutant (0.23±0.01 mM). At the time point of maximal biomass, the *ccpA* mutant had consumed 31% of the initial Gal, 5% more than that consumed by D39. Loss of CcpA did not affect the maximal Gal consumption rate (Table 2).

#### Growth and bioenergetic parameters

On Glc, the amount of biomass (g) formed per mol of substrate consumed or per mol of ATP (Y_ATP_) was higher in the CcpA-deficient strain. The same trend was observed for cultures growing on Gal: loss of CcpA resulted in higher biomass yields relative to substrate or ATP. The lower biomass yields in D39 indicate that, independently of the carbohydrate, CcpA directs the carbon source for catabolic processes. Assuming that all ATP is formed by substrate level phosphorylation, the higher ATP yields in conditions with higher acetate production were expected (Table 2). However, the parameters biomass yield and yield on biomass relative to ATP were lower on Glc than on Gal for both D39 and the *ccpA* mutant. These data indicate that Glc is a poorer substrate than Gal in bioenergetic terms, as more Glc is necessary to produce similar amounts of pneumococcal biomass.

In brief, when Glc was used as carbon source transcriptome and metabolic information correlated positively, but on Gal the fermentation end-products deviated from the predictions based on transcript levels suggesting regulation at other cellular levels.

### CcpA influences the intracellular levels of phosphorylated metabolites

**Figure 4:**
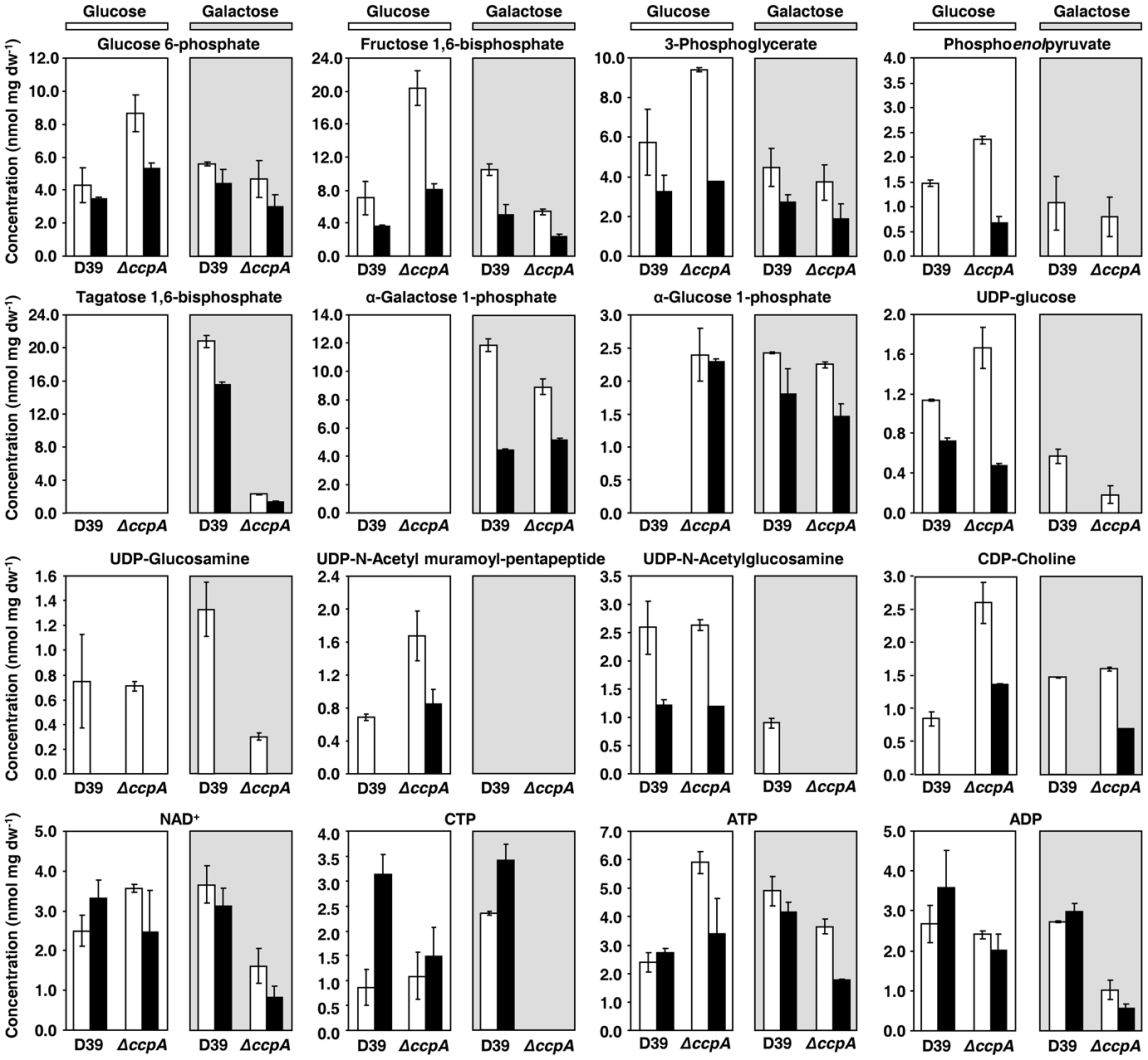
Effect of *ccpA* deletion on intracellular concentrations of phosphorylated metabolites during growth on Glc or Gal. Phosphorylated metabolites were measured by ^31^P-NMR in ethanol extracts of *S. pneumoniae* D39 and *ΔccpA* strains grown to mid-exponential (M, white bars, OD_600_ of 0.35±0.02) or transition-to-stationary phases (TS, black bars, OD_600_ of 1.3±0.1) of growth in CDM supplemented with 56±1 mM Glc (white background) or 57±1 mM Gal (light grey background). Phosphorylated metabolites measured in extracts comprised glycolytic metabolites, phosphorylated carbohydrate-specific metabolites, UDP-activated metabolites, and co-factors. The values are the mean of three independent experiments ± SD.

**Figure 5:**
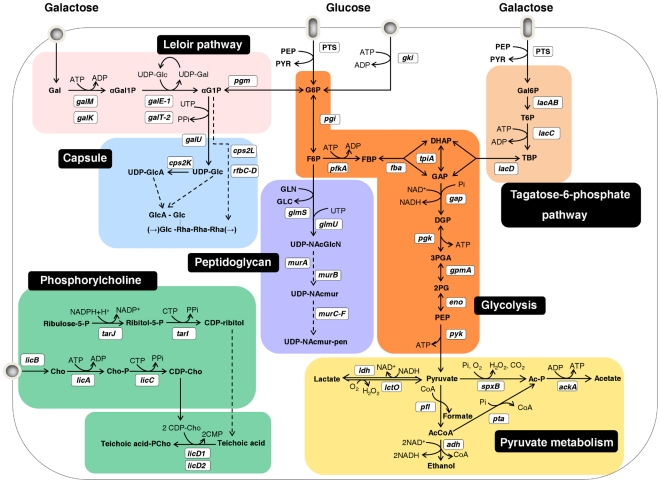
Schematic representation of central metabolic pathways in *S. pneumoniae* D39. Glc is oxidized to pyruvate via the Embden-Meyerhof-Parnas pathway (Glycolysis, orange box). Homolactic fermentation reduces pyruvate into lactate, whereas mixed-acid fermentation leads to other products, such as formate, acetate and ethanol (Pyruvate metabolism, yellow box). Gal is converted to G6P by the Leloir pathway (light pink box) and to dihydroxyacetone phosphate or glyceraldehyde 3-phosphate by the tagatose 6-phosphate pathway (light orange box). Pathways for capsule, peptidoglycan and phosphorylcholine biosynthesis are shown in light blue, light purple and light green boxes, respectively. Putative and functional characterized genes encoding depicted metabolic steps are shown in white boxes. Proposed pathways were reconstructed based on genome information (http://www.ncbi.nlm.nih.gov/genomes/lproks.cgi), literature and database surveys (KEGG, MetaCyc). Gene annotation downloaded from NCBI: *galM*, aldose 1-epimerase; *galK*, galactokinase; *galE-1*, UDP-glucose 4-epimerase; *galT-2*, galactose 1-phosphate uridylyltransferase; *pgm*, phosphoglucomutase/phosphomannomutase family protein; *galU*, UTP-glucose 1-phosphate uridylyltransferase; *cps2L*, glucose 1-phosphate thymidylyltransferase; *cps2K*, UDP-glucose 6-dehydrogenase, putative; *rfbC*, dTDP-4-dehydrorhamnose 3,5-epimerase, putative; *rfbB*, dTDP-glucose 4,6-dehydratase; *rfbD*, dTDP-4-dehydrorhamnose reductase; *gki*, glucokinase; *pgi*, glucose 6-phosphate isomerase; *pfkA*, 6-phosphofructokinase; *fba*, fructose bisphosphate aldolase; *tpiA*, triosephosphate isomerase; *gap*, glyceraldehyde-3-phosphate dehydrogenase; *pgk*, phosphogltcerate kinase; *gpmA*, phosphoglyceromutase; *eno*, phosphopyruvate hydratase; *pyk*, pyruvate kinase; *lacA*, galactose 6-phosphate isomerase subunit LacA; *lacB*, galactose 6-phosphate isomerase subunit LacB; *lacC*, tagatose 6-phosphate kinase; *lacD*, tagatose 1,6-diphosphate aldolase; *ldh*, L-lactate dehydrogenase; *lctO*, lactate oxidase; *spxB*, pyruvate oxidase; *ackA*, acetate kinase; *pta*, phosphotransacetylase; *pfl*, pyruvate formate-lyase; *adh* (spd_1834), bifunctional acetaldehyde-CoA/alcohol dehydrogenase; *glmS*, D-fructose 6-phosphate amidotransferase; *glmU*, UDP-N-acetylglucosamine pyrophosphorylase; *murA-1 and murA-2*, UDP-N-acetylglucosamine 1-carboxyvinyltransferase; *murB*, UDP-N-acetylenolpyruvoylglucosamine reductase; *murC*, UDP-N-acetylmuramate-L-alanine ligase; *murD*, UDP-N-acetylmuramoyl-L-alanyl-D-glutamate synthetase; *murE*, UDP-N-acetylmuramoylalanyl-D-glutamate-2,6-diaminopimelate ligase; *murF*, UDP-N-acetylmuramoylalanyl-D-glutamyl-2, 6-diaminopimelate–D-alanyl-D-alanyl ligase; *tarJ* (spd_1126), alcohol dehydrogenase, zinc-containing or TarJ; *tarI* (spd_1127), 2-C-methyl-D-erythritol 4-phosphate cytidylyltransferase or TarI; *licB*, protein LicB; *pck*, choline kinase or LicA; *licC*, CTP:phosphocholine cytidylyltransferase; *LicD1*, phosphotransferase LicD1; *LicD2*, phosphotransferase LicD2.

Inactivation of CcpA renders a strain with altered growth and fermentation profiles both on Glc and on Gal. In particular, the disparate substrate consumption rates and the fermentation end-product patterns, led us to surmise that levels of intracellular metabolites are also under the influence of CcpA. Intracellular metabolites were determined in ethanol extracts obtained as for transcriptome analyses (M and TS phases of growth) by targeted metabolomics using ^31^P-NMR (Fig. 4). In this way we were able to quantify intracellular concentrations of metabolites in central carbon utilization pathways (carbohydrate-specific pathways, glycolysis), catabolic precursors in the synthesis of surface structures (cell wall, capsule), and co-factors (Fig. 5), detailed below.

#### Glycolytic and carbohydrate-specific pathway intermediates

During growth on Glc the levels of the upper glycolytic metabolites glucose 6-phosphate (G6P) and FBP were higher in the *ccpA* mutant, independently of the phase of growth. Likewise, the lower glycolytic metabolites 3-phosphoglycerate (3-PGA) and phospho*enol*pyruvate (PEP) accumulated to higher concentrations in strain D39Δ*ccpA*. The size of the glycolytic pools showed a growth phase dependency: the concentrations of G6P, FBP, 3-PGA and PEP, were smaller in the TS phase. The decrease in the level of the lower glycolytic metabolites from M to TS phases of growth was unexpected and is contrary to the accumulation profile of 3-PGA and PEP in other *Streptococcaceae* species [19], [20], suggesting different regulation at the level of pyruvate kinase in *S. pneumoniae*. The concentrations of the glycolytic intermediates were also lower in the TS phase of growth when Gal was used as sole carbon source. In contrast to Glc, loss of CcpA on Gal resulted in slightly lower glycolytic pool levels. Furthermore, D39 and D39Δ*ccpA* cells growing on Gal accumulated tagatose 1,6-bisphosphate (TBP), α-galactose 1-phosphate (α-Gal1P) and α-glucose 1-phosphate (α-G1P), denoting simultaneous dissimilation of Gal via the tagatose 6-phosphate and Leloir pathways (Fig. 4 and 5). While deletion of *ccpA* had only a moderate negative effect on the accumulation of the Leloir intermediates (α-Gal1P and α-G1P), the concentration of TBP was 10-fold lower in D39Δ*ccpA*. This striking difference might be a direct consequence of the higher *lac* transcript levels in D39 (Table S1 and S4), since the overall Gal consumption flux was identical in both strains (Table 2). It should be noted that TBP and α-Gal1P are not detected during growth on Glc, whereas α-G1P, which is also the key precursor of structural polysaccharides (eg. capsule, teichoic acids) accumulated exclusively in the *ccpA* mutant.

#### NDP-activated intermediates in the synthesis of surface structures

NDP-activated intermediates are soluble precursors in the synthesis of several cell surface structures of utmost importance for virulence and pathogenesis [1], [2]. During growth on Glc, UDP-glucose, a precursor in the biosynthesis of the serotype 2 capsule and other structural polysaccharides, showed growth dependent accumulation in both strains, but the reduction from M to TS was more pronounced in the *ccpA* mutant. Furthermore, the accumulation profile of the soluble cell wall precursor UDP-N-acetylglucosamine was not changed due to inactivation of *ccpA*, whereas the concentration of UDP-N-acetylmuramoyl-pentapeptide was higher in D39Δ*ccpA*. Curiously, on Gal the soluble cell wall components were below detection limits (<0.5 mM), except for UDP-N-acetylglucosamine, which accumulated in *S. pneumoniae* D39 in MS growth. Accumulation of CDP-choline, the phosphorylcholine donor in the synthesis of teichoic acids, was higher in the *ccpA* mutant, decreasing in both strains from M to TS phases during growth on Glc. The growth dependent behaviour was also observed on Gal, but in this carbohydrate the levels of CDP-choline were approximately the same in D39 and D39Δ*ccpA*.

#### Concentrations of metabolic cofactors

CcpA-defective cells growing on Glc accumulated slightly higher amounts of NAD^+^, identical levels of CTP and ADP, and circa 2-fold higher amounts of ATP in MS growth (Fig. 4). In the TS phase of growth NAD^+^ and ATP were not significantly affected, ADP was slightly lower, and CTP was about half the concentration in the *ccpA* mutant. When Gal was used as substrate, the accumulated levels of NAD^+^, CTP, ATP and ADP were lower in the *ccpA* mutant both in M and TS phases of growth.

The metabolite profiles herein presented clearly support the operation of two routes for Gal catabolism and suggest a unique mechanism underlying the regulation of glycolysis in *S. pneumoniae*. CcpA influences the level of key phosphorylated metabolites (glycolytic metabolites, co-factors, etc) potentially involved in metabolic regulation. These findings extend beyond transcriptional regulation the role of CcpA in controlling cellular network properties. Furthermore, CcpA-dependent changes in cell wall and capsule UDP-activated precursors indicates a role of CcpA in the modulation of cell surface properties, known to be critical in virulence and pathogenesis.

### CcpA inactivation loosens capsule attachment to the cell wall

**Figure 6:**
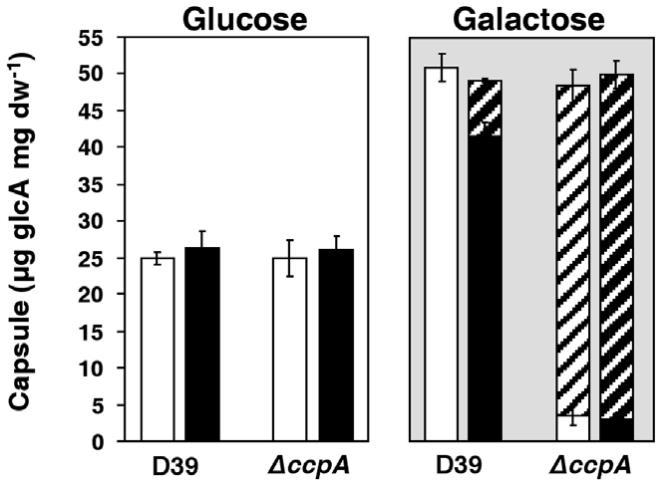
Amount of capsule polysaccharide in D39 and its isogenic *ccpA* mutant. Estimation of capsule was performed based on the determination of its glucuronic acid content in strains D39 and D39*ΔccpA* in mid-exponential (white bars, OD_600_ of 0.35±0.02) and transition-to-stationary (black bars, OD_600_ of 1.3±0.1) cultures grown in CDM containing 56±1 mM Glc (white background) or 57±1 mM Gal (light grey background). Hatched bars indicate loose capsule polysaccharide. All the determinations were done twice in two independent cultures and the values are means ± SD.

It has previously been reported that CcpA-deficient mutants show attenuated virulence, and this behaviour was associated with lower expression of genes committed to the synthesis of the pneumococcal capsule [11]. Our metabolome data showed significant differences in the pools of the capsule cytoplasmic precursors UDP-glucose and α-G1P. Therefore, we deemed it important to measure the amounts of capsule produced in D39 and its *ccpA* mutant (Fig. 6). Curiously, inactivation of *ccpA* did not affect the total amount of capsule either on Glc- or Gal-CDM. However, in Gal-containing medium the amount of capsule produced was higher for both strains. Furthermore, a major difference in Gal-CDM was the amount of loose capsule (not attached to the cell wall), which accounted for about 94% of the total polysaccharide in D39Δ*ccpA* (Fig. 6). In the parent strain, only a small fraction (∼13%) of loose capsule was detected, exclusively in the TS phase of growth.

Although the role of CcpA in the regulation of capsule production was not confirmed, our data show a function of CcpA in the association of capsule to the cell wall, at least during growth on Gal.

### CcpA affects the expression of many virulence genes

To further identify links between CcpA and virulence we compared all genes that have been linked to the virulence of this pathogen with our list of genes influenced by deletion of *ccpA* (Table S7). In addition, the *ccpA* microarray data were compared to the list of genes identified in the STM screen performed by Hava and Camilli [21]. Virulence genes affected by CcpA included classical virulence factors as well as many genes involved in amino acid and carbohydrate metabolism, transcriptional regulators, and genes with unknown function (Table S7).

Of the classical virulence factors, the Mn^2+^-dependent superoxide dismutase gene *sodA*, involved in virulence and protection against oxidative stress [22], was upregulated in the *ccpA* mutant, which fits well with lower H_2_O_2_ concentrations observed in this strain. Interestingly, two of the three neuraminidase genes, namely *nanA* and *nanB*, which have been extensively studied regarding their important role in pneumococcal virulence [15], were strongly upregulated in the *ccpA* mutant in all four conditions as well. Recently, NanA had been shown to, in conjunction with BgaA and StrH, two other exoglycosidases, protect *S. pneumoniae* from phagocytic killing through prevention of complement deposition [23]. *StrH* was affected only slightly in Glc_TS and Gal_TS, but *bgaA* was, like *nanA* and *nanB*, upregulated in the *ccpA* mutant in all four conditions, most strongly on Glc; importantly, *bgaA* was repressed in Glc compared to Gal (Table S4) only in the wild-type, which further supports its role in nasopharyngeal colonization. All these three genes, as well as *nanB*, might be directly affected by CcpA, since there is a predicted *cre* site in their upstream region or in the upstream region of a gene with which they seem to be co-regulated in the array analyses. Of a total of 79 virulence genes of which transcription is affected in the *ccpA* mutant, 35 are possibly regulated directly by CcpA (Table S7).

The *pcpA* gene, implicated in colonization/infection of the airways [24], showed glucose-dependent CcpA-mediated repression in D39. The *pcpA* gene is also regulated depending on the extracellular concentration of Mn^2+^ and Zn^2+^ through the action of PsaR [25], and activated by the nutritional regulator CodY [26]. It therefore seems to be regulated in a complex way in response to various different environmental stimuli.

Interestingly, genes involved in choline metabolism, namely the *licABC* and *tarJI* operons were upregulated in the *ccpA* mutant especially in Glc_TS. In agreement, the levels of CDP-choline were higher in the *ccpA* mutant (Fig. 4). Together the data suggest that *ccpA* could affect the level of choline in the pneumococcal cell wall and as a result the decoration of the pneumococcal cell wall with choline binding proteins, a class of virulence factors.

An interesting observation, which might be associated with virulence, was the strong activation by CcpA of two genes involved in cell wall synthesis (*glmS* and *murF*) only during growth on glucose. Moreover, the increased transcript level of *glmS* and *murF* in D39 on glucose might correlate with the high demand for synthesis of biomass as denoted by the high growth rate in this condition.

Of the transcriptional regulators identified as virulence genes, *comDE* was downregulated on Glc, whereas *TCS07* was upregulated in all conditions, which suggests that their regulons and their respective stimuli are connected to that of CcpA.

With a few exceptions, CcpA acts as a repressor of virulence gene expression in *S. pneumoniae*. Based on our data it is reasonable to assume that CcpA is one of the factors controlling the interaction of *S. pneumoniae* with its host during colonization and infection of the airways through regulation of the above mentioned key virulence genes.

## Discussion

**Figure 7:**
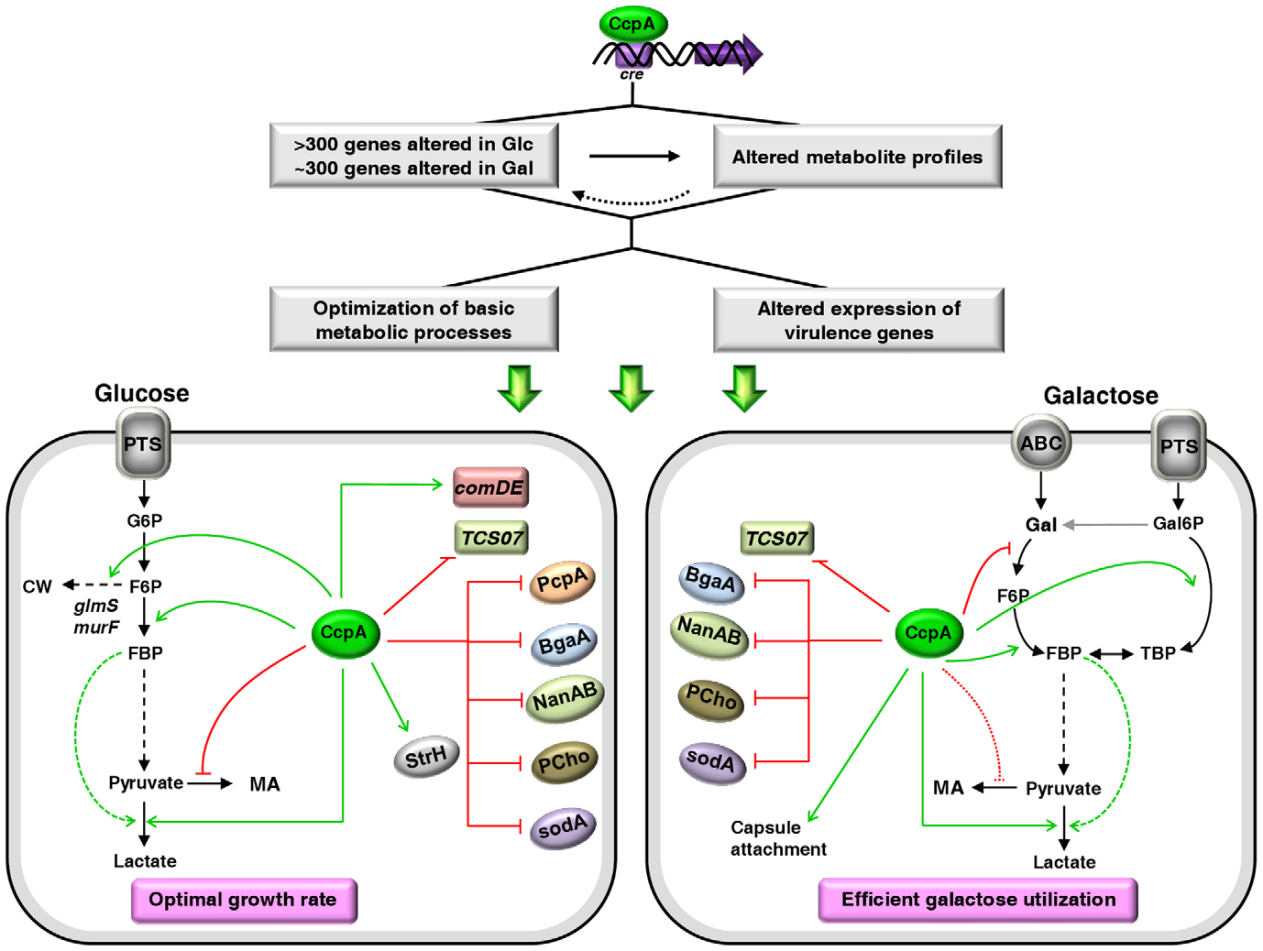
Model for CcpA regulation of basic metabolic processes and virulence factor expression in the presence of Glc or Gal in *S. pneumoniae*. Transport of carbohydrates through the multi-protein phospho*enol*pyruvate phosphotransferase system (PTS) ensures a typical PTS-mediated signal transduction pathway for CcpA regulation. CcpA-mediated regulation results in altered expression of genes involved in carbohydrate metabolism and classical virulence factors, including cell wall associated proteins, transcriptional regulators and phosphorylcholine. Repression and activation are represented by red and green arrows, respectively. Dashed lines indicate metabolic regulation. G6P, Glucose 6-phosphate; F6P, fructose 6-phosphate; CW, cell wall; MA, mixed-acid fermentation; PcpA, choline binding protein PcpA; BgaA, beta-galactosidaseA; NanAB, neuraminidases A and B; PCho, Phosphorylcholine; SodA, superoxide dismutase, manganese-dependent; StrH, β-N-acetylhexosaminidase; TCS07 and ComDE, two-component systems.

It is well established that bacteria adapt to fluctuating environments by altering expression of genes involved in basic metabolic processes. In the Firmicutes, the pleiotropic transcriptional regulator CcpA is recognized as a key player in carbon catabolite control, *i.e.*, the regulatory phenomena that allow bacteria to selectively use substrates for growth (for reviews see [3], [4]). Importantly, in the last decade an increasing number of studies have exposed the role of CcpA in the regulation of virulence in Gram-positive pathogens [7], [10], [11], [27]–[29]. In the human pathogen *S. pneumoniae*, regulation of carbohydrate-specific catabolic pathways as well as modulation of virulence factors by CcpA has been reported [10]–[12]. However, a comprehensive study of the role of CcpA in the physiology of *S. pneumoniae* was not undertaken so far. In a recent study, *ccpA* was found to interact with 64 other genes by Tn-seq transposon mutagenesis; based on the high number of genetic interactions these authors suggested that CcpA appears to be a master regulator in *S. pneumoniae* [30]. In this work, we used genome-wide transcriptome analyses and metabolite profiling to study in depth the impact of CcpA on pneumococcal metabolism and virulence. Our data show that beyond controlling carbohydrate transport and metabolic genes, CcpA is involved in various other cellular processes, including traits connected to virulence and pathogenesis (Fig. 7). Therefore, the role of CcpA as a master regulator in *S. pneumoniae* is undoubtedly demonstrated by the results in this study.

The CcpA regulon was substantial on Glc, the “preferred” carbohydrate of many Firmicutes, which supports high pneumococcal growth rate (Fig. 3), but remarkably this was also the case in the slowly metabolized Gal, a carbohydrate generally recognized as non-repressing. In *B. subtilis*, besides Glc several other PTS-transported carbohydrates are capable of eliciting CcpA-mediated catabolite repression [31]. In *S. mutans*, the transport of Gal is exclusively mediated via PTS systems [9], but curiously the CcpA regulon on Gal was markedly smaller than on Glc [7], which is in contrast to our observations in *S. pneumoniae*. Also noticeable is the pronounced difference in the amount of genes influenced by CcpA in *S. mutans* (up to 9%) and *S. pneumoniae* (up to 19%), which despite their genetic relatedness apparently have evolved distinct regulatory networks. In *S. mutants,* catabolite repression can be independent of CcpA and requires specific components of carbohydrate-PTS permeases [9]. A reasonable explanation lies in the nutritional diversity encountered by the two streptococci in their respective ecological niches.

In this study, CcpA ensures fast growth on Glc, but not on Gal, as the growth rate on the latter carbohydrate is basically independent of this pleiotropic regulator. In *S. pneumoniae* the routes for Gal catabolism have not been biochemically characterized, but complete Leloir and tagatose 6-phosphate pathways can be deduced from pneumococcal genome sequence data (http://www.ncbi.nlm.nih.gov/genome/lproks.cgi). We propose that both pathways are operative during growth on Gal. The presence of the intermediates α-Gal1P (Leloir) and TBP (tagatose 6-phosphate), as well as the elevated expression levels of the genes *galM, galKT-2* (Leloir), and *lacABCD* (tagatose 6-phosphate), fully support this claim. Interestingly, *lacFE* are also induced by Gal, suggesting a role of the pneumococcal lactose-PTS in Gal uptake as reported for *S. mutans* and *L. lactis* [9], [32]. The uptake of Gal via a PTS system would ensure a typical PTS-mediated signal transduction pathway for CcpA regulation and is in agreement with the large CcpA regulon determined during growth on Gal. On this carbohydrate, CcpA regulation seems to comply with the essence of catabolite control, which is to coordinate metabolic operation ensuring a maximum profit for growth. Activation by CcpA of the PTS-dependent route (tagatose 6-phosphate pathway) for Gal degradation, while repressing the Leloir genes, is in agreement with that principle. Furthermore, activation of the glycolytic genes *pfk* and *pyk*, and *ldh* indicate that CcpA's role is to enable maximal Gal catabolism. In light of our results, an answer to the question as to why substrate consumption and growth rates on Gal are not stimulated by CcpA is difficult to put forward. Of note in Gal-grown D39 cells, is the elevated concentration of TBP (10-fold higher than in D39Δ*ccpA*), which might be toxic for the cells. Indeed, unrestricted accumulation of phosphorylated metabolites has previously been evoked to explain negative effects on growth rate [33], [34]. Also surprising was the pronounced shift to mixed acid fermentation in strain D39, since CcpA represses genes involved in that type of metabolism. However, Gal-induced alleviation of the CcpA-mediated repression might partially explain the observed behaviour (Table S1). Indeed, higher expression of *pfl* and *pfl-AE* allow a more efficient competition with lactate dehydrogenase for the common substrate pyruvate, consequently increasing the flux to mixed acid products, including acetate. Production of this organic acid is accompanied by ATP formation, which is certainly an advantage during the fermentation of Gal, as the glycolytic ATP generation is limited by the substrate consumption rate (three times lower than that of Glc). Shifts to mixed acid fermentation as a means to counteract for ATP production during growth on slowly metabolized carbon sources or limiting carbohydrate concentrations carbohydrates are well documented in *Streptococacceae* [35], [36]. In view of the data it is reasonable to assume that Gal-dependent regulatory mechanisms at the translational, posttranslational and/or metabolic levels overlay the transcriptional direct control by CcpA. A good example is the Gal-dependent activation of the Leloir pathway, which genes are in all instances under CcpA repression. The lack of correlation between phenotypic behaviours and changes in transcription levels is not unprecedented [37], [38], and most likely derives from the feedback regulation of metabolism on all regulatory layers [13].

In this work we show that CcpA is crucial in the control of central physiological processes in *S. pneumoniae*, and thus most likely influences the ability to persist and proliferate in the host. Considering our data showing Glc- and Gal-dependent regulation it is tempting to hypothesize that in the specific host microenvironments basic metabolic processes are tuned by CcpA to maximize cellular profit (Fig. 7). In this light, the attenuated virulence shown by *ccpA* mutants in mouse models of pneumococcal colonization and infection [10], [11] can plausibly be attributed to decreased fitness. However, this hypothesis might not depict the full picture, as we discovered that CcpA directly or indirectly influences the transcript level of a number of canonical virulence factors (Fig. 7). It is interesting to note that among the influenced genes several encode moonlighting proteins, with roles in host-pathogen interaction and basic metabolic processes. A good example is provided by BgaA, an exoglycosidase involved in adherence to epithelial cells [15], and recently found to be important in basal growth [30], which is repressed by CcpA as determined by us and others [10], [12]. Such findings certainly reinforce the subtle link between basic physiology and virulence in *S. pneumoniae*.

Involvement of CcpA in the control of classical virulence factors is a recurrent observation in *Streptococacceae* [7], [8], [10], [11], [30]. In a previous report, significant repression of the capsular polysaccharide biosynthesis locus (*cps*) was observed in a pneumococcal *ccpA* mutant [11]. Except for a negligible effect on *rfbCBD* genes in the transition-to-stationary phase of Glc-grown cells, we have not found significantly differentially expressed *cps* genes in any other tested conditions. Furthermore, the amount of capsule produced was not influenced by CcpA (Fig. 6). Accordingly, Camilli and co-workers did not observe a connection between CcpA and *cps* expression [10], [30]. Our data unequivocally show that the production of capsule is influenced by the carbon source, since the amount of polysaccharide was doubled in Gal-grown cells in a CcpA-independent manner. In view that Gal is a prevailing carbohydrate in nasopharyngeal secretions [15], [39], this finding is relevant in terms of host-pathogen interactions, since a relatively thick capsule is essential to evade the immune system, and thus, for the pneumococcus to persist in the host [40]. Curiously, on this carbohydrate the attachment of the capsular polysaccharide to the pneumococcal cell wall was substantially altered in the presence of CcpA.

Our transcriptome analyses revealed that genes involved in the synthesis of phosphorylcholine are under the control of CcpA. At the metabolic level, the lower concentration of CDP-choline (activated choline precursor) is in full agreement with the transcript levels of the biosynthetic genes. The tight control exerted by CcpA on the availability of CDP-choline might provide a means to influence the association of capsule, proteins and other molecules to the cell wall. Indeed, binding of macromolecules to the cell wall is conditioned by the decoration of the wall polysaccharides (teichoic acids and lipoteichoic acids) [41], [42], which in *S. pneumoniae* contain residues of phosphorylcholine [42]. Using proteomics others have shown that loss of CcpA caused changes in the amounts of several cell wall-associated proteins [10]. A good correlation between our expression data and the wall fraction protein levels was observed for some proteins [10], but for other proteins no significant regulation at the transcriptional level was detected. Although we cannot rule out the possibility that this is due to interspecies variation or translational and post-translation regulation, in light of our results it is reasonable to speculate that, rather than altering the protein level, CcpA is somehow affecting the tethering of the proteins to the cell wall, as we showed for capsule attachment. CcpA-dependent effects on the levels of several NDP-activated precursors of wall polysaccharides (Fig. 4) further strengthen the role of CcpA in shaping the cell wall decoration. This unforeseen function of CcpA might be crucial in the host-pneumococcal interaction, which is heavily dependent on cell wall phosphorylcholine and associated proteins [2]. Future studies on cell-surface proteome should be performed to address this issue in more detail.

Of the promoters differentially regulated in the *ccpA* mutant *circa* 42% are presumably subject to direct regulation by CcpA, as denoted by the presence of a *cre* sequence. This value is in good agreement with the 38% previously determined using the Tn-seq method to evaluate genetic interactions [30]. Still, a large percentage of the regulated promoters lack a canonical *cre* site. CcpA influenced the transcript level of 22 other transcriptional regulators, and thereby indirectly affects genes that are under the control of these regulators. Interestingly, we found that CcpA-responsive genes are also part of other regulatory networks. About 15 genes, mainly involved in amino acid and carbohydrate metabolism, but also the virulence gene *pcpA*, are commonly regulated by the nutritional regulator CodY and CcpA [26]. Additionally, CcpA was found to share many gene targets with two-component systems regulatory networks, including those of CiaRH [43], TCS04 [44], TCS06 [45], TCS08 [46], and TCS09 [47]. Recent studies in *S. pyogenes* have highlighted the importance of a combination of independent transcriptional regulators to coordinate gene expression during infection [28], [48]. In this light, our data opens new avenues to explore how *S. pneumoniae* virulence is shaped by co-regulation of independent transcriptional regulators.

Factors controlling the interaction between pathogens and their host are of utmost importance for *in vivo* fitness and persistence in adverse host niches. Previously, the role of the transcriptional regulator CcpA in the physiology of the pneumococcus has been underappreciated. In this work we resorted to a combined transcriptome and metabolite profiling approach to demonstrate that CcpA is a master regulator involved in varied cellular processes in *S. pneumoniae*. We have ascertained that *S. pneumoniae* optimizes basic metabolic processes, modulates expression of virulence factors and most likely tunes association of macromolecules with the cell wall in a CcpA-mediated manner. The observed carbohydrate-dependency of the CcpA regulatory network indicates a key role in adjusting to specific nutritional cues within host niches. Exploring the unveiled links between CcpA and other regulators will certainly contribute to an improved understanding on the ability of *S. pneumoniae* to colonize and cause disease. The insights gained thus far from this comprehensive analysis foresee CcpA as a key factor in the interaction between *S. pneumoniae* and its host.

## Materials and Methods

### Bacterial strains, media and growth conditions

Strains and plasmids used in this study are shown in Table 1. *S. pneumoniae* strains were grown as standing cultures without aeration in M17 broth (Difco™) containing Glc 0.5% (w/v) or on Glc-M17 (0.5% w/v) agar plates with 1% (v/v) defibrinated sheep blood (Johnny Rottier) at 37°C. When appropriate 150 µg ml^−1^ spectinomycin, 2.5 µg ml^−1^ chloramphenicol, 0.25 µg ml^−1^ erythromycin or 15 µg ml^−1^ trimethoprim were added to the medium. Capsule quantification, transcriptomic and metabolic experiments were performed with cells grown in chemically defined medium (CDM) [19], supplemented with disodium β-glycerophosphate (21 g L^−1^), sodium pyruvate (0.01% w/v), choline (0.001% w/v) and cysteine (0.4 g L^−1^) in static flasks at 37°C without pH control (initial pH 6.5). Glc (56±1 mM) or Gal (57±1 mM) was used as the carbon source. For complementation studies, nisin was added at time zero. Growth of the cultures was monitored by measuring optical density at 600 nm every hour. Maximum specific growth rates (µ_max_) were calculated through linear regressions of the plots of ln (OD_600_) versus time during the exponential growth phase.

### Molecular techniques

Chromosomal DNA isolation was performed according to the procedure of Johansen and Kibenich [49]. Plasmid isolation was carried out using the plasmid isolation kit from Roche. *Phusion* DNA polymerase (Roche), T4 DNA ligase (Roche) and restriction enzymes (Fermentas) were used according to the recommendations described by the suppliers. Purification of the PCR products was performed using the High pure PCR product purification kit from Roche. Purified PCR products or recombinant plasmids were introduced into *S. pneumoniae* by transformation as described in Kloosterman *et al.* [50]. Positive transformants were selected on Glc-M17 agar with the appropriate antibiotic and confirmed by PCR and sequencing. *L. lactis* MG1363 was transformed with plasmid DNA by electroporation according to Holo and Nes [51].

### Construction of a ccpA mutant of S. pneumoniae D39

Oligonucleotide primers used in this study are listed in Table 1. In *S. pneumoniae* D39, *ccpA* forms a monocistronic operon, flanked upstream by a well defined promoter region and downstream by a strong terminator (predicted ΔG° = −13.8 kcal mol^−1^). The *ccpA* deletion was accomplished by allelic replacement mutagenesis as follows: the up- (547 bp) and downstream (517 bp) regions of the *ccpA* gene (1010 bp) were PCR-amplified from D39 chromosomal DNA using the primer pairs ccpA_D39_KO-1/ccpA_D39_KO-2 (spec) and ccpA_D39_KO-3 (spec)/ccpA_D39_KO-4, respectively. The resulting PCR products were, by means of overlap extension PCR [52], fused to a Spc^R^ gene (1032 bp, amplified with primers Spec_Fp and Spec_Rp from plasmid pORI38) using the primers ccpA_D39_KO-1 and ccpA_D39_KO-4. The Δ*ccpA::spc* PCR product (2096 bp) obtained in this way was transformed to *S. pneumoniae* D39, giving rise to the *ccpA* deletion mutant (D39Δ*ccpA* in Table 1). Replacement of the *ccpA* gene with the *spc* gene and correct integration of the insert were confirmed by PCR and sequencing (Service XS). To rule out the possibility of suppressor mutations arising with the deletion of *ccpA*, the transformation efficiency of the Δ*ccpA::spc* PCR product was compared with that of the SPD_0786*::spc* (SPD_0786 insertion-deletion) PCR product. SPD_0786 is a non-essential transcription factor, which disruption does not affect growth of *S. pneumoniae* in CDM (Kloosterman *et al.*, unpublished results). A similar number of transformants per ng of DNA (CFU ng^−1^) was obtained, strongly suggesting that our *ccpA* strain contains no suppressor mutations.

### Transformation frequency assay

For the transformation frequency assay the 2096-bp fragment comprising the Δ*ccpA::spc* region was PCR-amplified from the chromosomal DNA of the *ccpA* mutant strain using the primer pairs ccpA_D39_KO_1 and ccpA_D39_KO_4. Similarly, a fragment (2725-bp) encompassing the SPD_0786*::spc* region of a D39 SPD_0786*::spc* strain was amplified using appropriate primer pairs. An identical concentration of each product was used to transform strain D39 as described above. Spectinomycin resistant colonies were selected and counted for each transformation and the number of colonies was compared to evaluate the transformation frequency.

### Construction of the ccpA complementation strain

A *ccpA* complementation strain was constructed using the NICE system improved for *S. pneumoniae* [50]. In this arrangement the NisRK two-component system, coded by the *nisRK* genes, activate the expression of the *nisA* promoter in the presence of nisin. The procedure to construct the *ccpA* complementation strain is described as follows. The *ccpA* mutant strain (D39Δ*ccpA*) was transformed with chromosomal DNA from D39*nisRK*, a stable derivative of *S. pneumoniae* D39 that harbours the *nisRK* genes and a Trim^R^ gene in the *bgaA* locus. Trimethoprim resistant clones (D39Δ*ccpAnisRK* in Table 1) were selected and the presence of both the *ccpA* deletion and the *nisRK* genes was confirmed by PCR. For the construction of the plasmid harbouring *ccpA* under the control of the nisin-indusible promoter *nisA* (pNZ[*ccpA*] in Table 1), the *ccpA* gene was PCR-amplified from D39 chromosomal DNA using the primers ccpA_D39-1 and ccpA_D39-2 and cloned as a *BsaI/XbaI* fragment in *NcoI/XbaI* digested pNG8048E. *L. lactis* MG1363 was used as the cloning host. The ccpA complementation strain (D39*ΔccpAnisRK*pNZ[*ccpA*] in Table 1) was constructed by transformation of the D39*ΔccpAnisRK* strain with plasmid pNZ[*ccpA*] selecting for chloramphenicol resistant clones. As a control D39Δ*ccpAnisRK* strain was also transformed with the empty plasmid pNZ8048. Strains were confirmed by PCR.

### Transcriptome analysis

*S. pneumoniae* D39 and the isogenic *ccpA* mutant were compared by transcriptome analysis using whole-genome *S. pneumoniae* DNA microarrays, representing all TIGR4 ORFs, as well as R6 and D39 ORFs that are not in TIGR4 [53]. See http://www.ncbi.nlm.nih.gov/geo/query/acc.cgi?acc=GPL11484, for a description of all amplicons and oligo's present on the DNA microarray. The two strains were grown as described above in three biologically independent experiments and harvested at mid-exponential (OD_600_ of 0.35±0.02) and transition-to-stationary (OD_600_ of 1.3±0.1) phases of growth (Fig. S1). mRNA isolation, synthesis of cDNA, labelling of cDNA, hybridization, scanning of the slides and the processing and analysis of the data was carried out as described before [53]. Genes were considered to have significantly altered expression when the bayesian *p*-value was <0.001, the false discovery rate (FDR) was <0.01 and the ratio (signal Δ*ccpA*/signal D39) was >1.5 or <0.66, but >2 or <0.5 in at least one of the four conditions. For the Gal_TS comparison, data were derived from two independent hybridizations and a Bayesian *p*-value <0.01, FDR <0.08 was applied. To exploit the data obtained in this way, also an *in silico* comparison was done between Glc and Gal, both for the M and TS phases and also in both D39 wild type as well as *ΔccpA*. To this end, raw signals (*i.e.* slide images) of the Glc experiments were compared with the corresponding signals of the Gal experiments and analyzed as described above. The microarray data have been deposited at GEO under accession number GSE31819.

### *Cre* site prediction

Using the *L. lactis* consensus sequence (WGWAARCGYTWWMA, [6], a *cre* site prediction was performed in D39, using Genome2D [17]. Subsequently, genes with a *cre* site in their promoter region that were differentially expressed in the DNA microarray analyses of the *ccpA* mutant were used to built a weight matrix, which was subsequently used to search the genome again using Genome2D [17], using a cut-off score of 9.9. In a similar way, the weight matrix of the RegPrecise (http://regprecise.lbl.gov/RegPrecise/) prediction was used. The resulting predictions (Table S6) were compiled together with the original prediction of RegPrecise and a prediction done in a recent publication [30]. The resulting list of 340 genes with putative *cre* sites was compared with the genes that were differentially expressed in the arrays. Of these 340 genes with *cre* sites, 115 matched with the list of genes differentially expressed in the *ccpA* mutant in at least one condition. After re-analysis of these matches with an online *cre* site prediction tool (http://molgen51.biol.rug.nl/websoftware/ppp/ppp_start.php), 90 genes were left that contain a putative *cre* site in their promoter region, or downstream of the translational start.

### Real-time quantitative RT-PCR

Cell samples obtained as described above for transcriptome analyses using DNA microarrays were treated with RNase-free DNase I (Fermentas, St. Leon-Rot, Germany) for 60 min at 37°C in DNaseI buffer (10 mmol l^−1^ Tris-HCl (pH 7.5), 2.5 mmol l^−1^ MgCl_2_, 0.1 mmol l^−1^ CaCl_2_). Samples were purified with the Roche RNA isolation kit. Reverse transcription was performed on 5 µg of total RNA using the same method as used during the microarray procedure. Quantification of specific cDNA of spd_0420 (*pfl*), spd_0667 (*sodA*), spd_0790 (*pyk*), spd_1053 (*lacE*), spd_1078 (*ldh*), spd_1504 (*nanA*) and spd_1634 (*galK*) was performed on an CFX96 Real-Time PCR System (BioRad, Hercules, CA) using Maxima SYBR Green qPCR Master Mix (Fermentas, St. Leon-Rot, Germany) on 5 ng cDNA template in a 20 µl reaction volume. Primers used are listed in Table 1. The qRT-PCR data were normalized to the level of *metG* (spd_0689) cDNA using the 2^−ΔΔC^_T_ method [54]. The data are averages of four repeats.

### Quantification of fermentation products during growth

Strains were grown as described above. Culture samples (2 ml) were taken in mid-exponential and transition-to-stationary phases of growth, centrifuged (16,000×*g*, 2 min, 4°C), filtered (Millex-GN 0,22 µm filters) and the supernatant solutions were stored at −20°C until analysis by high performance liquid chromatography (HPLC). Prior to analysis, samples were allowed to thaw at room temperature. Glc or Gal and end-products were quantified in an HPLC apparatus equipped with a refractive index detector (Shodex RI-101, Showa Denko K. K.) using an HPX-87H anion exchange column (Bio-Rad Laboratories Inc.) at 60°C, with 5 mM H_2_SO_4_ as the elution fluid and a flow rate of 0.5 ml min^−1^. Alternatively, quantification of metabolites in the supernatant solutions was performed by ^1^H-NMR in a Bruker Avance II 500 MHz spectrometer (Bruker BioSpin GmbH). Formic acid (sodium salt) was added to the samples and used as an internal concentration standard. The ATP yield was calculated as the ratio of ATP produced to Glc or Gal consumed. The global yields of ATP were calculated from the fermentation products determined at the time-point of growth arrest assuming that all ATP was synthesized by substrate-level phosphorylation. A factor of 0.39, determined from a DW (mg ml^−1^) versus OD_600_ curve, was used to convert OD_600_ into dry weight (mg biomass ml^−1^).Hydrogen peroxide was quantified in fresh supernatant solutions using the Amplex® Red hydrogen Peroxide/Peroxidase assay kit from Invitrogen.

### Determination of hydrogen peroxide (H_2_O_2_)

Strains were grown in chemically defined medium (CDM) as described above. Culture samples of 1-ml were harvested in mid-exponential and transition-to-stationary phases of growth, centrifuged (16,000×*g*, 2 min, 4°C) and filtered (Millex-GN 0,2 µm filters). The Amplex® Red hydrogen Peroxide/Peroxidase assay kit from Invitrogen was used to quantify the hydrogen peroxide contents in the fresh supernatant solutions.

### Cold ethanol extractions and determination of intracellular metabolites by ^31^P-NMR

The ethanol extracts were prepared as described previously by Ramos *et al*. [55]. For each extract the volume of cells harvested from mid-exponential (OD_600_ of 0.35±0.02) and transition-to-stationary (OD_600_ of 1.3±0.1) cultures corresponded to approximately 14 mg of protein and the centrifugation was performed at 4°C, 6,000×*g*, during 3 min. The dried extract was dissolved in 1 ml of deuterated water (pH 6.5 or pH 5.5) containing 5 mM EDTA and stored at −20°C for further analysis by ^31^P-NMR. This methodology was successfully used to measure phosphorylated metabolites in *L. lactis* [55]. In *S. pneumoniae* ethanol extracts the following phosphorylated metabolites were detected: G6P, FBP, 3-PGA, PEP, α-Gal1P, α-G1P, TBP, UDP-Glc, UDP-N-acetylmuramoyl-pentapeptide, UDP-N-acetylglucosamine, UDP-glucosamine, CDP-choline, NAD^+^, CTP, ATP and ADP. A small peak due to fructose 6-phosphate was also detected, but its quantification hampered by strong overlapping with a resonance due to the medium component β-glycerophosphate. A weak resonance due to dihydroxyacetone phosphate was present, but reliable integration was not possible. The concentration limit for detection/quantification of metabolites in ethanol extracts by ^31^P-NMR was approximately 0.4–0.5 mM. Resonances were assigned by addition of pure compounds to the extracts or on the basis of comparison with a previous study [55]. Concentrations were calculated from the areas of the resonances in ^31^P-spectra by comparison with the area of a resonance due to methylphosphonic acid (Aldrich), added as an internal standard, and after application of an appropriate factor for correcting saturation of resonances. The reported concentration values for intracellular phosphorylated compounds are averages of three independent growth experiments.

^31^P-NMR spectra were recorded using a selective probe head (^31^P-SEX) at 37°C on a Bruker AVANCE II 500 MHz spectrometer (Bruker BioSpin GmbH) by using standard Bruker pulse programs. Spectra were referenced to the resonance of external 85% H_3_PO_4_, designated at 0 ppm.

### Capsular polysaccharide preparation and quantification of the glucuronic acid content

Samples for the determination of the capsular glucuronic acid amounts were prepared as follows: cells grown in CDM and harvested in mid-exponential (OD_600_ of 0.35±0.02) and transition-to-stationary (OD_600_ of 1.3±0.1) phases of growth were centrifuged (6,000×*g*, 3–7 min, 4°C), resuspended in PBS and pelleted at 3000×*g*, 4°C, for 20 min. The pellet was resuspended in 500 µL of 150 mM Tris-HCl (pH 7.0) and 1 mM MgSO_4_ and treated as described elsewhere [56]. The supernatants derived from the two centrifugations referred before were pooled and treated with 20% (w/v) trichloroacetic acid for protein precipitation. After 2 h of incubation in an ice bath proteins were pelleted and the exopolysaccharides were precipitated with cold ethanol as in Ramos *et al.* [55]. The glucuronic acid of capsule attached or loosely attached (lost in centrifugations to the supernatants) to the cell wall was quantified by the method for quantitative determination of uronic acids as described by Blumenkrantz *et al.* [57].

## Supporting Information

Figure S1 **Growth profiles of D39 wild-type and the isogenic** ***ccpA*** **mutant on Glc and Gal.** (A) Schematic overview of the *ccpA* gene and its flanking genes and the genetic replacement of the *ccpA* gene with a spectinomycin marker in strain D39. Hooked arrow, putative promoter; lollipop, putative terminator; black area, *ccpA* gene replaced with a spectinomycin cassette; numbers inside the genes, D39 SPD numbers; arrows and numbers above the figure indicate primers used to construct the D39Δ*ccpA* mutant; zoomed area (inset), putative *cre* box, −35 and −10 promoter regions; the number of bp spacing these regions are subscripted after {n}. (B and C) Growth of strains D39 (▪), D39Δ*ccpA* (▴) and D39Δ*ccpAnisRK*pNZ[*ccpA*] (○) in CDM containing 1% Glc (B) or 1% Gal (C) at 37°C in rubber-stoppered static bottles without pH control (initial pH 6.5); growth curves as in Figure 1, except that logarithmic scale was used for the y-axis. The growth of the complemented strain was performed without nisin in the medium. Optical densities at 600 nm (OD_600_) were measured hourly. Each point of the growth curves is an average of at least three independent experiments and the error was in all cases below 15%. The growth rates for each strain are also indicated and the values shown are averages ± SD. The arrows indicate the mid-exponential and transition-to-stationary time-points at which D39 and D39Δ*ccpA* samples were withdrawn for transcriptomic and metabolic profiling analysis.
(TIF)

Table S1 **Overview of all significantly up- or downregulated genes as determined by the DNA microarray analysis of D39Δ** ***ccpA*** **compared to D39 wild-type (yellow columns).** Only genes were included that have a ratio of (D39Δ*ccpA*/D39 wt) >1.5 or <0.66, and >2.0 or <0.5 in at least one of the four conditions tested (See also Materials and Methods). In case genes were also significantly up- or downregulated (according to the same criteria) in the *in silico* comparison of Glc compared to Gal, which was done for both the M and TS phases and for both D39 wt and the D39Δ*ccpA*, these data were also given (green columns, ratios are Glc/Gal). In addition, putative *cre* sites are given in columns AG to AJ. For more details, see column headers. Glc = glucose, Gal = galactose, wt = D39 wild-type, *ccpA* = D39Δ*ccpA*, M = mid-exponential growth phase, TS = transition-to-stationary growth phase, bayes p. = bayesian p-value, FDR = false discovery rate, COG = cluster of orthologous groups.
(XLS)

Table S2 **Expression ratio of selected genes linked to metabolic and virulence pathways (** ***spd_0420 (pfl), spd_0667 (sodA), spd_0790 (pyk), spd_1053 (lacE), spd_1078 (ldh), spd_1504 (nanA)*** **and** ***spd_1634 (galK)*** **as determined by qRT-PCR in exponentially and transition to stationary phases of growth.** Samples were obtained as described for the microarray experiments and the experiments performed as in Materials and Methods. The qRT-PCR data were normalized to the level of *metG* (*spd_0689*) cDNA and the data are averages of four repeats.
(XLS)

Table S3 **Subtable abstracted from Table S1, giving only the genes in COG [G] (Carbohydrate transport and metabolism).** Putative substrates for carbohydrate transporters regulated by CcpA are indicated as well. ABC, ATP binding cassette transporter; PTS, phosphoenolpyruvate:carbohydrate phosphotransferase system.
(XLS)

Table S4 **Overview of all significantly (according to the criteria as outlined in the legend for Table S1) up- or downregulated genes as determined by the** ***in silico*** **DNA microarray analysis of the signals in Glc compared to the signals in Gal (Glc/Gal, green columns).** In case genes were also significantly up- or downregulated (according to the same criteria) in the D39Δ*ccpA* versus D39 wild-type comparison, these data were also given (yellow columns). As in Table S1, putative *cre* sites are given as well. For more details, see column headers and legend Table S1.
(XLS)

Table S5 **List of genes with putative** ***cre*** **sites, as predicted and compiled in this study as described in detail in the Materials and Methods.**
(XLS)

Table S6 **List of genes with putative** ***cre*** **sites that were not significantly affected in the DNA microarray analyses of D39Δ** ***ccpA*** **compared to D39 wild-type.**
(XLS)

Table S7 **Subtable abstracted from Table S1, giving the virulence genes regulated by CcpA. Gene names and references are given as well.**
(XLS)
