# Supplementary figures and images for: CcpA Ensures Optimal Metabolic Fitness of Streptococcus pneumoniae
Source: PLoS One. 2011 Oct 21;6(10):e26707. doi: 10.1371/journal.pone.0026707 (PMC3198803; doi:10.1371/journal.pone.0026707)

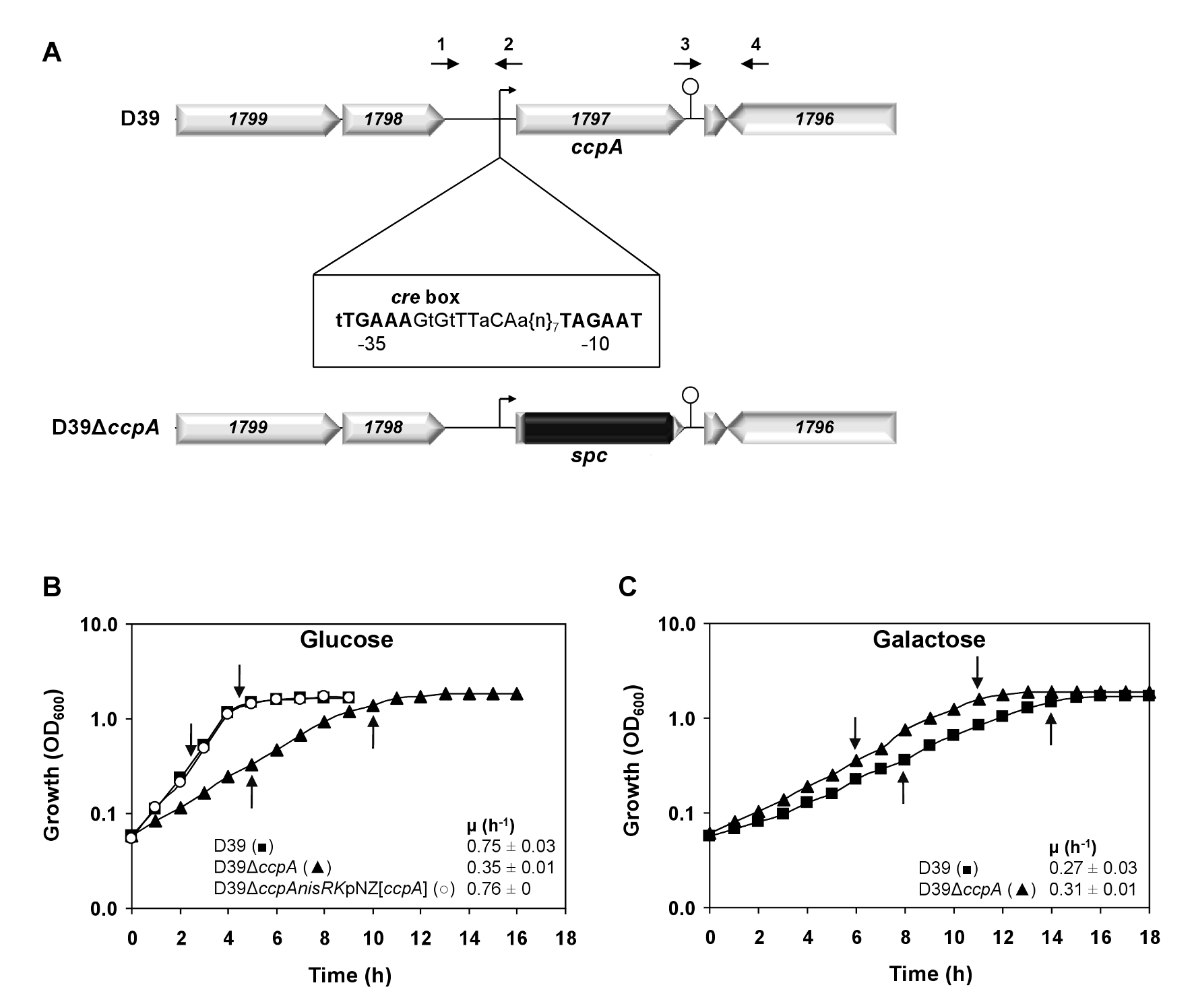

Supplement: Figure S1 — Growth profiles of D39 wild-type and the isogenic ccpA mutant on Glc and Gal. (A) Schematic overview of the ccpA gene and its flanking genes and the genetic replacement of the ccpA gene with a spectinomycin marker in strain D39. Hooked arrow, putative promoter; lollipop, putative terminator; black area, ccpA gene replaced with a spectinomycin cassette; numbers inside the genes, D39 SPD numbers; arrows and numbers above the figure indicate primers used to construct the D39ΔccpA mutant; zoomed area (inset), putative cre box, −35 and −10 promoter regions; the number of bp spacing these regions are subscripted after {n}. (B and C) Growth of strains D39 (▪), D39ΔccpA (▴) and D39ΔccpAnisRKpNZ[ccpA] (○) in CDM containing 1% Glc (B) or 1% Gal (C) at 37°C in rubber-stoppered static bottles without pH control (initial pH 6.5); growth curves as in Figure 1, except that logarithmic scale was used for the y-axis. The growth of the complemented strain was performed without nisin in the medium. Optical densities at 600 nm (OD600) were measured hourly. Each point of the growth curves is an average of at least three independent experiments and the error was in all cases below 15%. The growth rates for each strain are also indicated and the values shown are averages ± SD. The arrows indicate the mid-exponential and transition-to-stationary time-points at which D39 and D39ΔccpA samples were withdrawn for transcriptomic and metabolic profiling analysis. (TIF) [file pone.0026707.s001.tif]
